# Hepatitis C prevalence and risk factors in Georgia, 2015: setting a baseline for elimination

**DOI:** 10.1186/s12889-019-6784-3

**Published:** 2019-05-10

**Authors:** Liesl M. Hagan, Ana Kasradze, Stephanie J. Salyer, Amiran Gamkrelidze, Maia Alkhazashvili, Gvantsa Chanturia, Nazibrola Chitadze, Roena Sukhiashvili, Marina Shakhnazarova, Steven Russell, Curtis Blanton, Giorgi Kuchukhidze, Davit Baliashvili, Susan Hariri, Stephen Ko, Paata Imnadze, Jan Drobeniuc, Juliette Morgan, Francisco Averhoff

**Affiliations:** 1 Division of Viral Hepatitis, Centers for Disease Control and Prevention, Atlanta, GA USA; 2 National Center for Disease Control and Public Health, Tbilisi, Georgia; 3 Division of Global Health Protection, Centers for Disease Control and Prevention, Atlanta, GA USA; 4 School of Public Health, Boston University, Boston, MA USA; 5 Global Disease Detection – South Caucasus Regional Center, Centers for Disease Control and Prevention, Tbilisi, Georgia

**Keywords:** Hepatitis C virus, HCV, HCV elimination, Georgia, HCV prevention, Global health security

## Abstract

**Background:**

The country of Georgia launched the world’s first Hepatitis C Virus (HCV) Elimination Program in 2015 and set a 90% prevalence reduction goal for 2020. We conducted a nationally representative HCV seroprevalence survey to establish baseline prevalence to measure progress toward elimination over time.

**Methods:**

A cross-sectional seroprevalence survey was conducted in 2015 among adults aged ≥18 years using a stratified, multi-stage cluster design (*n* = 7000). Questionnaire variables included demographic, medical, and behavioral risk characteristics and HCV-related knowledge. Blood specimens were tested for antibodies to HCV (anti-HCV) and HCV RNA. Frequencies were computed for HCV prevalence, risk factors, and HCV-related knowledge. Associations between anti-HCV status and potential risk factors were calculated using logistic regression.

**Results:**

National anti-HCV seroprevalence in Georgia was 7.7% (95% confidence interval (CI) = 6.7, 8.9); HCV RNA prevalence was 5.4% (95% CI = 4.6, 6.4). Testing anti-HCV+ was significantly associated with male sex, unemployment, urban residence, history of injection drug use (IDU), incarceration, blood transfusion, tattoos, frequent dental cleanings, medical injections, dialysis, and multiple lifetime sexual partners. History of IDU (adjusted odds ratio (AOR) = 21.4, 95% CI = 12.3, 37.4) and blood transfusion (AOR = 4.5, 95% CI = 2.8, 7.2) were independently, significantly associated with testing anti-HCV+ after controlling for sex, age, urban vs. rural residence, and history of incarceration. Among anti-HCV+ participants, 64.0% were unaware of their HCV status, and 46.7% did not report IDU or blood transfusion as a risk factor.

**Conclusions:**

Georgia has a high HCV burden, and a majority of infected persons are unaware of their status. Ensuring a safe blood supply, implementing innovative screening strategies beyond a risk-based approach, and intensifying prevention efforts among persons who inject drugs are necessary steps to reach Georgia’s HCV elimination goal.

## Background

Globally, there are an estimated 71 million people living with hepatitis C virus (HCV) infection and 411,000 HCV-attributable deaths annually [1]. HCV is bloodborne and transmitted most often through unsterile medical equipment, infected blood and tissue used for medical procedures, and shared drug injection equipment. HCV infection often progresses asymptomatically for 20–30 years, and most HCV-related deaths result from liver cirrhosis or hepatocellular carcinoma decades after the incident HCV infection [2–6]. HCV accounts for an estimated 27% of cirrhosis and 25% of hepatocellular carcinoma cases worldwide [7].

Georgia is an Eastern European, middle-income country with 3.7 million residents [8]. A 2002 survey in the capital city of Tbilisi found that 6.7% of the general population and 70.4% of persons who inject drugs had antibodies to HCV (anti-HCV, evidence of past or current HCV infection) [9], suggesting that HCV prevalence in Georgia could be among the highest globally. In 2015, Georgia launched the world’s first HCV elimination program, aiming to provide universal access to curative, direct-acting antiviral (DAA) treatment at no cost to patients, and to implement nationwide prevention measures to curb transmission [10]. Existing prevalence data have been instrumental in engaging the government’s strong support to combat the country’s HCV burden, but are outdated and not nationally representative. Data documenting updated nationwide HCV prevalence and risk factors for infection are necessary to effectively plan treatment and prevention services supporting Georgia’s HCV elimination goals, and to establish a baseline to track progress toward elimination over time.

This paper presents the results of the first nationally representative HCV seroprevalence survey in Georgia, conducted in 2015 by Georgia’s National Center for Disease Control and Public Health (NCDC) in collaboration with the United States Centers for Disease Control and Prevention (CDC). The Georgian government is using these results to plan and implement HCV surveillance, education, prevention, screening, care, and treatment efforts. A follow-up survey is planned to assess the impact of interventions designed to achieve HCV elimination. In addition, planning and conducting this national serosurvey provided an important opportunity to strengthen the public health capacity in Georgia and thereby enhance global health security.

## Methods

### Sample design

A cross-sectional, nationally representative seroprevalence survey was conducted in Georgia from May–August 2015 among adults aged ≥18 years using a stratified, multi-stage cluster design. A sample size of 7000 was calculated based on estimated 6.7% anti-HCV seroprevalence [9], a design effect of 2, and an anticipated 70% response rate. The sample was designed to yield a nationwide HCV prevalence estimate, independent prevalence estimates in six pre-selected major cities, and in urban vs. rural areas overall. Region-level estimates were also calculated where sample size was sufficient.

The country was divided into 16 mutually exclusive sampling strata (six major cities and ten regions). Strata defined by a region contained both urban and rural areas, but excluded any of the six major cities that lie within the region’s boundaries. The occupied territories of Abkhazia and South Ossetia were excluded.

The sampling frame was a full list of Georgia’s 9503 census tracts, provided by Georgia’s National Statistics Office (GeoStat). These census tracts served as the primary sampling units (clusters) within each stratum. Equal size tracts were assumed since a size measure was not available during sample selection. To reach a sample size of 7000, 280 clusters were selected across the 16 strata, 25 households were selected within each cluster, and one participant was selected from each household. The six major cities were oversampled (120 clusters) to increase precision of point estimates. The remaining 160 clusters were allocated to the ten regions proportionally based on their population size. The specific clusters sampled within each stratum were randomly selected from the list provided by GeoStat.

Within each selected cluster, 25 households were systematically selected using an algorithm based on the cluster’s total number of year-round households. Within each household, the Kish method was applied to randomly select one adult for participation [11]. Household members aged ≥18 years who had spent the previous night in the house were eligible; temporary guests and household members living outside the home were excluded. If the selected individual was unavailable, two revisit attempts were made; no replacement participants were selected if the individual was unavailable after revisits or refused participation.

### Data collection

Interviewers trained and supervised by NCDC and CDC epidemiologists administered a structured questionnaire to participants who provided informed consent. The questionnaire was given verbally in participants’ preferred language (Kartuli/Georgian, Russian, Armenian, or Azeri) and included demographics, medical history, lifestyle/behavioral history, and knowledge of HCV. Data were entered into hand-held electronic devices in real time and uploaded to a secure database. Survey questions were vetted by local staff to ensure cultural appropriateness and suitability for laypersons with a primary school education. Field and laboratory procedures, questionnaires, and informed consent forms were piloted in rural and urban areas.

Nurse-phlebotomists collected 10 mL blood specimens from consenting participants. Specimens were centrifuged in the field, transported to public health laboratories for processing and testing, and stored at the Georgian National Reference Laboratory in Tbilisi. Each participant’s specimen and questionnaire data were linked using a unique barcode. Personal identifying information was obtained strictly to report laboratory test results to participants, and was removed before epidemiologic analysis.

### Laboratory methods

Anti-HCV and HCV RNA testing were performed in Georgian public health laboratories. CDC laboratory staff monitored protocols and processes for quality assurance/quality control. All specimens were tested for anti-HCV by enzyme-immunoassay (HCV Ab v4.0 EIA *IVD*, Dia.Pro. Diagnostic Bioprobes Srl, Italy). Anti-HCV-positive specimens were tested for HCV RNA (Sacace™ HCV Real-TM Qual, Sacace Biotechnologies Srl, Italy). Anti-HCV-positive/RNA-negative specimens underwent confirmatory anti-HCV testing using a third generation line immunoassay (INNO-LIA™ HCV Score, *IVD*, Innogenetics N.V., Belgium); specimens that tested positive or indeterminate for anti-HCV in confirmatory testing were re-tested for HCV RNA in the CDC Division of Viral Hepatitis Assay Development and Diagnostic Reference Laboratory in Atlanta, Georgia, USA using a highly sensitive, FDA-licensed assay (COBAS Ampliprep/COBAS Taqman® CAP/CTM v2.0, *IVD,* Roche, Indianapolis, IN, USA); specimens testing HCV RNA negative in the CDC laboratory were re-tested for anti-HCV using the FDA-licensed VITROS Immunodiagnostic System (aHCV, *IVD*, Ortho Clinical Diagnostics, Raritan, NJ, USA) to identify false positives. All specimens were tested for hepatitis B virus and human immunodeficiency virus; results are not reported in this manuscript.

Laboratory test results were reported securely to participants via the Georgian Post; to receive the mailing, participants were required to present a national identification card matching the name of the addressee. Participants with a positive HCV RNA test received written instructions for accessing Georgia’s national HCV treatment program in the same mailing.

### Statistical methods

Statistical analyses were conducted using SAS 9.3 (Cary, North Carolina, USA). Data were weighted based on probability of selection at cluster, household, and individual levels, and adjusted to represent Georgia’s national population by sex, age, and geographic distribution using 2014 census data. Analyses used complex survey procedures accounting for stratification, clustering, and unequal sample weights. Variance was calculated using Taylor series linearization.

Anti-HCV prevalence was calculated by demographic characteristics and potential HCV risk factors; weighted prevalence estimates and 95% confidence intervals (CI) are presented. Bivariate associations between anti-HCV positivity and demographic and risk factor characteristics were examined using Rao-Scott chi-square tests; associations were considered significant when *p* < .05. An unconditional logistic regression model was utilized to explore the relationship between anti-HCV positivity and multiple risk factors that were significantly associated with anti-HCV status in bivariate analyses. Backward elimination was used to reduce the full model; variables were retained if the Wald F test p < .05. Variables without significant, independent associations with anti-HCV positivity were retained as confounders if they changed parameter estimates for other significant predictor variables in the main effects model by ≥10%. All potential pairwise interactions in the final model were examined and considered significant if the Wald F test p < .05. The final model was assessed for multicollinearity. Odds ratios and 95% CI are presented. Weighted percentages and 95% CI were computed for HCV knowledge variables. Unweighted percentages were computed for HCV treatment history variables.

This survey was determined to be a routine public health activity for public health surveillance by CDC’s Human Subjects Research Office and therefore judged to not involve human subjects research.

## Results

Of 7000 adults selected, 6296 (89.9%) consented to participate, and 6014 (85.9%) provided both questionnaire responses and a blood specimen. Response rates exceeded 70% in all strata. Three specimens were hemolyzed during processing, and one returned inconclusive anti-HCV results. Demographic analyses include all 6296 participants; HCV-specific analyses include the 6010 participants who provided both questionnaire responses and a blood specimen yielding interpretable serologic test results.

### Participant demographics and exposures

Participants’ median age was 45 years; 53.8% were female, and 56.7% lived in urban areas (Table 1). 90.9% had completed secondary school or higher, and 19.5% were unemployed. Approximately two-thirds (64.0%) reported an annual household income less than the national average (12,268 Georgian Lari/$5254 US dollars) [12] .

**Table 1:** Demographic characteristics and reported exposures among survey participants, Georgia HCV serosurvey, 2015

Characteristic	n	Weighted % (95% CI)
Total Sample	6296	100.0
Sex		
Female	3868	53.8 (52.0, 55.5)
Male	2428	46.2 (44.5, 48.0)
Missing	0	
Age		
18–29	1115	19.4 (18.2, 20.7)
30–39	1177	19.4 (17.9, 20.9)
40–49	1070	18.6 (17.2, 20.0)
50–59	1140	16.5 (15.4, 17.7)
≥ 60	1790	26.1 (24.5, 27.8)
Missing	4	
Geography		
Urban	3350	56.7 (52.7, 60.6)
Rural	2946	43.3 (39.4, 47.3)
Missing	0	
Employment status		
Employed	2120	37.8 (35.6, 39.9)
Student	172	3.6 (2.9, 4.4)
Homemaker	1483	19.1 (17.7, 20.6)
Retired	1405	20.0 (18.7, 21.5)
Unemployed (able to work)	1110	19.5 (18.0, 21.1)
Missing	6	
Highest level of education completed		
Completed less than elementary/primary school	43	0.7 (0.5, 1.1)
Completed elementary/primary school	612	8.5 (7.3, 9.8)
Completed secondary school	2567	40.2 (38.1, 42.3)
Completed professional/technical school	1157	16.6 (15.3, 18.0)
Completed university/college or higher	1912	34.0 (31.6, 36.4)
Missing	5	
Yearly household income		
≤ 6000 GEL/year (≤ 4400 USD)	2867	45.6 (43.0, 48.3)
6001–12,000 GEL/year (4400–6800 USD)	953	18.5 (16.8, 20.3)
12,001–24,000 GEL/year (6800–13,600 USD)	724	12.6 (11.3, 13.9)
> 24,000 GEL/year (> 13,600 USD)	1339	23.3 (21.1, 25.8)
Missing	413	
Ever injected drugs		
Yes	208	4.2 (3.5, 5.2)
No	6042	95.8 (94.8, 96.5)
Missing	46	
Ever incarcerated		
Yes	240	4.6 (3.8, 5.7)
No	6037	95.4 (94.3, 96.2)
Missing	19	
Have any tattoos		
Yes	639	12.2 (10.9, 13.7)
No	5645	87.8 (86.3, 89.1)
Missing	12	
Ever received a blood transfusion		
Yes	459	7.0 (6.1, 7.9)
No	5828	93.0 (92.1, 93.9)
Missing	9	
Ever received kidney dialysis		
Yes	17	0.3 (0.2, 0.6)
No	6255	99.7 (99.4, 99.8)
Missing	24	
Number of medical injections received in last 6 months		
0	3857	62.8 (60.7, 64.8)
1	557	9.5 (8.4, 10.7)
> 1	1701	27.8 (26.0, 29.6)
Missing	181	
Frequency of dental cleanings		
Twice/year	199	4.4 (3.6, 5.3)
Once/year	491	9.0 (7.8, 10.2)
Less than once/year	1170	20.3 (18.5, 22.3)
Never	4370	66.3 (64.0, 68.5)
Missing	66	
Number of lifetime sexual partners		
0–2	4232	75.0 (73.1, 76.8)
> 2	1026	25.0 (23.2, 26.9)
Missing	1038	
Men who have sex with men (MSM)		
Yes	0	0
No	2185	90.0
Missing	243	10.0

When asked about risk factors for HCV infection, 4.2% reported a history of injection drug use (IDU), 7.0% reported receiving a blood transfusion, and < 1% reported receiving dialysis; 4.6% reported a history of incarceration, and 12.2% reported having at least one tattoo. None identified as men who have sex with men (MSM), and 25.0% reported having > 2 lifetime sexual partners.

### HCV prevalence

**Fig. 1:**
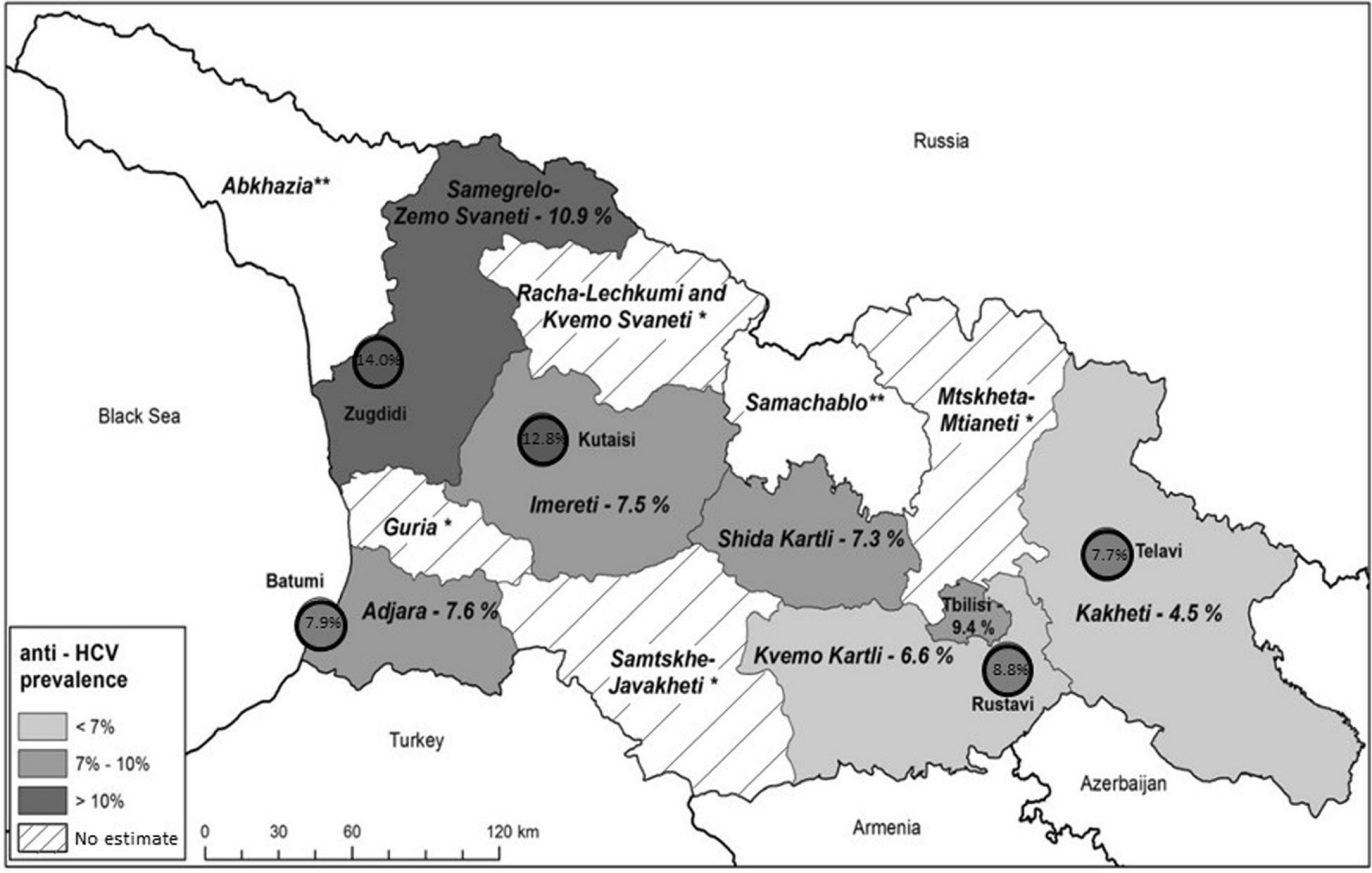
Anti-HCV prevalence in major cities and regions of Georgia. The highest regional anti-HCV prevalence was found in Samegrelo-Zemo Svaneti region in northwest Georgia (10.9%), particularly in the city of Zugdidi (14.0%, nearly double the national prevalence of 7.7%). In general, anti-HCV prevalence was higher in cities than in the surrounding rural areas. [Notes: *Anti-HCV prevalence estimates were not calculated for Guria region, Mtskheta-Mtianeti region, Racha-Lechkumi/Kvemo Svaneti region, or Samtskhe-Javakheti region due to insufficient sample size. **The occupied territories of Abkhazia and Samachablo (South Ossetia) were not included in the survey]

**Fig. 2:**
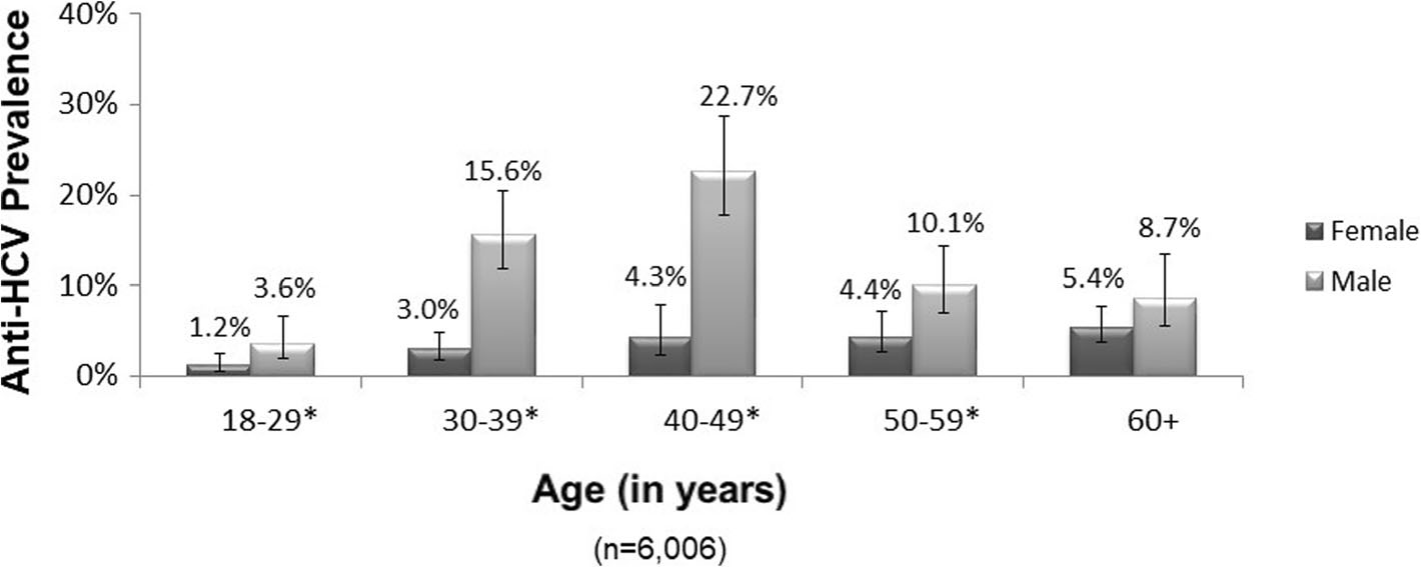
Anti-HCV prevalence by age and sex. Anti-HCV prevalence was approximately three times higher among men vs. women overall (12.1% vs. 3.8%) and varied by age; among men, prevalence peaked at 22.7% in the 40–49 age group, while it increased steadily with age among women to a maximum of 5.4% among those ≥60 years of age. [Note: *Differences in anti-HCV prevalence between male and female respondents were statistically significant in asterisked categories using Rao-Scott Chi-square tests (*p* < 0.05)]

Of the 6010 participants providing a usable blood specimen, 433 (7.7, 95% CI = 6.7, 8.9) tested anti-HCV positive, and 311 (5.4, 95% CI = 4.6, 6.4) tested HCV RNA positive (indicating chronic infection). Anti-HCV prevalence was higher in urban vs. rural areas (9.5% vs. 5.4%, *p* < 0.0001) (Table 2); the highest regional prevalence was in Samegrelo-Zemo Svaneti region in northwest Georgia (10.9%), particularly in the city of Zugdidi (14.0%) (Fig. 1). Anti-HCV prevalence was approximately three times higher among men vs. women (12.1% vs. 3.8%, p < 0.0001) and varied by age (Table 2); among men, prevalence peaked at 22.7% in the 40–49 age group, while it increased steadily with age among women to a maximum of 5.4% among those ≥60 years of age (Fig. 2).

**Table 2:** Anti-HCV prevalence by demographic and exposure subgroup in unadjusted and adjusted models, Georgia HCV serosurvey, 2015

Characteristic		Anti-HCV Prevalence		Unadjusted Models		Final Adjusted Model	
	Total n	n	Weighted % (95% CI)	Crude OR (95% CI)	*p-value*	Adjusted OR (95% CI)	p-value
Demographics							
Sex							
Female	3671	145	3.8 (3.0, 4.9)	1			
Male	2339	288	12.1 (10.2, 14.3)	3.5 (2.5, 4.8)	< 0.0001		
Missing	0						
Age							
18–29	1063	23	2.4 (1.5, 4.0)	1			
30–39	1140	94	8.8 (6.8, 11.3)	3.9 (2.2, 6.8)	< 0.0001		
40–49	1026	128	14.0 (11.1, 17.6)	6.5 (3.9, 11.1)	< 0.0001		
50–59	1096	79	7.0 (5.2, 9.5)	3.0 (1.6, 5.8)	0.0006		
60+	1681	109	6.7 (5.0, 9.0)	2.9 (1.6, 5.4)	0.0007		
Missing	4						
Geography							
Urban	3155	290	9.5 (8.0, 11.4)	1.8 (1.4, 2.5)	< 0.0001		
Rural	2855	143	5.4 (4.4, 6.6)	1			
Missing	0						
Employment Status							
Employed/student/	4939	286	5.9 (5.0, 7.1)	1			
homemaker/unpaid							
worker/retired							
Unemployed*	1065	147	15.0 (12.3, 18.1)	2.8 (2.1, 3.7)	< 0.0001		
Missing	6						
Exposures							
Ever injected drugs							
Yes	205	150	66.5 (56.0, 75.6)	37.6 (23.5, 60.0)	< 0.0001	21.4 (12.3, 37.4)	< 0.0001
No	5762	283	5.0 (4.3, 5.9)	1			
Missing	43						
Ever incarcerated							
Yes	236	98	42.0 (32.8, 51.7)	11.3 (7.5, 17.1)	< 0.0001		
No	5757	335	6.0 (5.1, 7.0)	1			
Missing	17						
Have any tattoos							
Yes	626	104	16.2 (12.2, 21.1)	2.8 (1.9, 4.0)	< 0.0001		
No	5372	329	6.5 (5.5, 7.6)	1			
Missing	12						
Number of medical injections in last 6 months							
0	3656	233	6.7 (5.6, 7.9)	1			
1	541	40	6.6 (4.3, 10.2)	0.99 (0.60, 1.65)	0.98		
> 1	1648	144	9.5 (7.5, 12.1)	1.48 (1.10, 1.99)	0.01		
Missing	165						
Ever received a blood transfusion							
Yes	447	69	21.4 (15.6, 28.5)	3.8 (2.6, 5.5)	< 0.0001	4.5 (2.8, 7.2)	< 0.0001
No	5554	364	6.7 (5.8, 7.7)	1			
Missing	9						
Received a blood transfusion before or after 1997							
Before 1997	225	36	25.3 (16.2, 37.3)	1.6 (0.7, 3.7)	0.27		
In or after 1997	222	33	17.4 (10.7, 27.1)	1			
Ever received kidney dialysis							
Yes	17	3	27.6 (7.9, 62.9)	4.6 (1.0, 20.4)	0.04		
No	5972	430	7.7 (6.7, 8.8)	1			
Missing	21						
Frequency of dental cleanings							
Twice/year	193	15	15.0 (8.1, 26.2)	2.1 (1.1, 4.4)	0.04		
Once/year	478	27	6.7 (3.9, 11.5)	0.9 (0.5, 1.6)	0.66		
Less than once/year	1108	84	6.9 (5.0, 9.4)	0.9 (0.6, 1.3)	0.58		
Never	4173	304	7.6 (6.5, 8.8)	1			
Missing	58						
Number of lifetime sexual partners							
0–2	4020	157	3.8 (3.0, 4.7)	1			
> 2	991	129	11.9 (9.1, 15.4)	3.4 (2.4, 5.0)	< 0.0001		
Missing	999						

Note: Anti-HCV related analyses include only participants who submitted both questionnaire data and a usable blood specimen (*n* = 6010)

*Unemployed includes those able or unable to work

### Factors associated with HCV infection

In bivariate analysis, anti-HCV positivity was significantly associated with male sex, unemployment, and urban residence, as well as history of IDU, incarceration, blood transfusion, tattoos, frequent dental cleanings, medical injections, dialysis, and having multiple lifetime sexual partners (Table 2). Among participants who reported a history of blood transfusion, no significant difference in anti-HCV prevalence was detected between those who reported receiving a transfusion before vs. in/after 1997 (when Georgia began testing donated blood for HCV) (Table 2). Other medical and community exposures including hospitalization, surgery, body piercings, and manicures/pedicures were not significantly associated with anti-HCV positivity (data not shown).

In the adjusted model, history of IDU (adjusted odds ratio (AOR) = 21.4, 95% CI = 12.3, 37.4) and receipt of a blood transfusion at any date (AOR = 4.5, 95% CI = 2.8, 7.2) were the only risk factors that were significantly, independently associated with anti-HCV positivity, controlling for sex, age, urban vs. rural residence, and history of incarceration (Table 2). [Note: A dichotomous blood transfusion variable (ever vs. never received transfusion) was used in the multivariate model.] There were no significant interactions in the final model.

Of the 433 anti-HCV positive participants, 38.2% reported IDU, and 19.7% reported receiving a blood transfusion. Nearly half of anti-HCV positive participants (46.7%) did not report either of these risk factors. Overall, 66.5% of anti-HCV positive participants were male, and 43.4% were ≥ age 50. The sex and age breakdown was similar among anti-HCV positive participants reporting a blood transfusion (63.2% male and 55.7% ≥ age 50) and among anti-HCV positive participants who did not report either IDU or history of blood transfusion (60.6% male and 46.2% ≥ age 50). Anti-HCV positive participants reporting IDU were mostly male (98.3%) and concentrated in the 30–49 age range (70.0%), with 16.3% ≥ age 50.

### HCV diagnosis and treatment

**Fig. 3:**
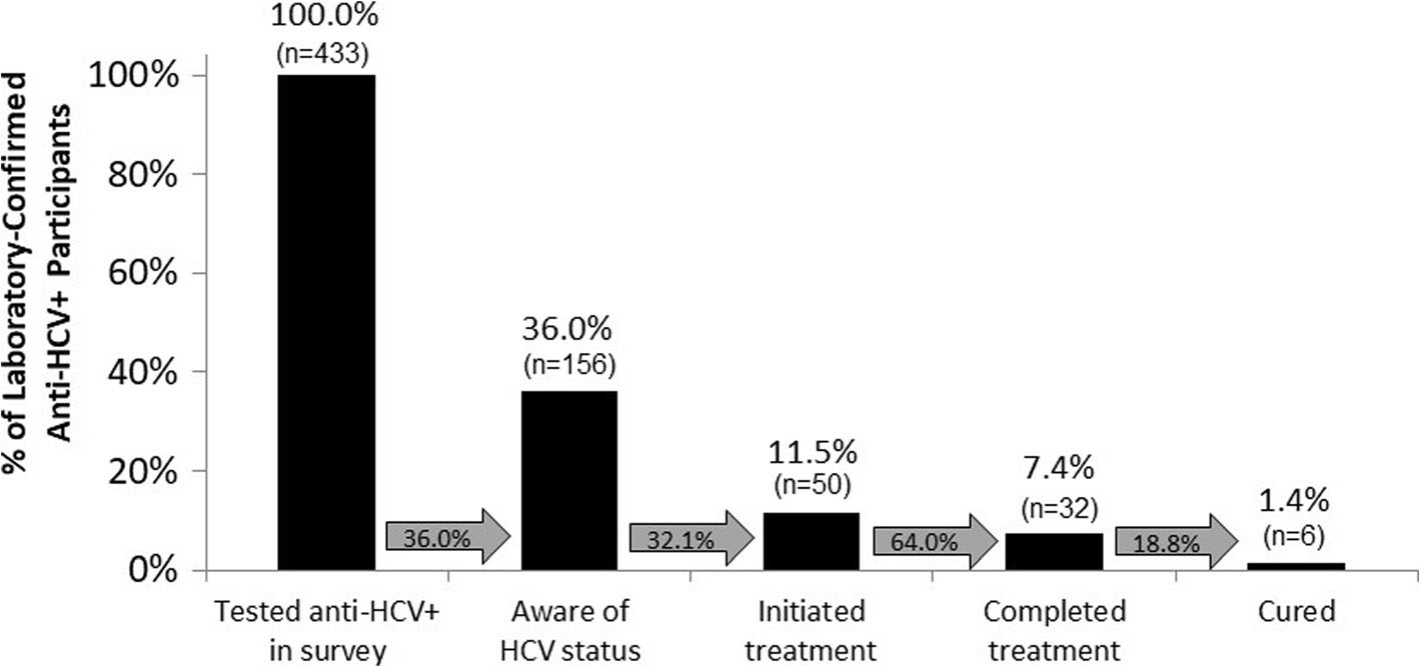
Self-reported cascade of HCV care among laboratory-confirmed anti-HCV positive participants. Among the 433 survey participants who tested anti-HCV positive, 156 (36.0%) already knew their HCV status prior to the survey. Among participants aware of their HCV infection, 50 (32.1%) reported initiating treatment prior to the survey, 32 (64.0%) of those who began treatment reported completing it, and 6 (18.8%) of those who completed treatment reported being cured

Among the 433 participants who tested anti-HCV positive, 156 (36.0%) already knew their HCV status prior to the survey. Awareness of HCV status was more likely among anti-HCV positive participants reporting IDU compared to those not reporting IDU (55.3% vs. 28.5%, *p* = 0·0002). Among participants aware of their HCV infection, 50 (32.1%) reported initiating treatment prior to the survey, 32 (64.0%) of those who reported initiating treatment reported completing it, and 6 (18.8%) of those who reported completing treatment reported being cured (Fig. 3). A cross-check of self-reports against laboratory test results revealed that 14 participants reporting treatment completion tested HCV RNA negative (more than twice the number who reported being cured); however only three of the six who reported a cure actually tested HCV RNA negative.

Among anti-HCV positive participants aware of their infection and reporting no treatment, reasons cited for non-treatment included lack of treatment availability (56.6%), high cost (33.0%), and anticipated side effects (12.3%).

### HCV-related knowledge

A majority of participants (56.1%) were aware that HCV can be transmitted through exposure to infected blood; when asked about specific transmission modes, 52.3% identified sharing needles/syringes, 43.6% identified sharing household objects that have had contact with blood, and 31.9% identified sexual contact as possible HCV transmission modes. More than half of participants (57.2%) were aware that HCV can be asymptomatic, and 70.5% knew that HCV is treatable. HCV-related knowledge was higher among participants who were anti-HCV positive, and highest specifically among anti-HCV positive participants reporting a history of IDU (Table 3).

**Table 3:** HCV-related knowledge by anti-HCV status and reported IDU history, Georgia HCV serosurvey, 2015

	All participants		Anti-HCV+ participants		Anti-HCV+ participants reporting IDU	
	n	Weighted % (95% CI)	n	Weighted % (95% CI)	n	Weighted % (95% CI)
Aware that HCV can be asymptomatic	2458	57.2 (54.9, 59.4)	260	76.4 (69.9, 81.9)	120	83.7 (74.2, 90.2)
Aware that HCV can be treated	3041	70.5 (68.5, 72.5)	287	83.6 (77.5, 88.3)	130	89.0 (80.1, 94.2)
HCV can be transmitted by						
Blood	3295	56.1 (53.9, 58.3)	295	71.1 (64.8, 76.8)	136	89.0 (80.2, 94.1)
Sharing needles or syringes	3056	52.3 (50.0, 54.6)	278	67.1 (60.4, 73.2)	128	87.2 (78.8, 92.6)
Sharing household objects like	2582	43.6 (41.1, 46.1)	249	58.6 (51.7, 65.1)	114	73.4 (62.4, 82.1)
razors or toothbrushes						
Sexual contact	1875	31.9 (30.1, 33.7)	165	41.4 (35.0, 48.1)	83	57.5 (46.3, 67.9)

When asked what sources they trust for information about their health, 35.8% of participants identified doctors and other healthcare workers, and 34.0% identified television. Other information sources including the internet, family/friends, medical literature, newspapers, radio, brochures/fliers, pharmacists, and billboards, were each cited as trustworthy by fewer than 15% of participants (data not shown). Participants were able to select multiple responses to this question.

## Discussion

HCV elimination has garnered increasing international support since the development of curative HCV drugs in recent years, resulting in the World Health Organization’s worldwide HCV elimination plan, the European Union HCV Policy Summit commitment to elimination, and individual elimination programs in Georgia, Australia, Iceland, the Cherokee Nation in the United States, and other areas [13–17]. Georgia was the first country to undertake HCV elimination and has set ambitious targets including a 90% reduction in chronic HCV prevalence by 2020 [10, 18].

This survey confirms that Georgia has a high burden of HCV infection and identifies risk factors that will be essential to address in Georgia’s HCV elimination strategy. Applying the 5.4% HCV RNA prevalence found in this survey to Georgia’s adult population of 2.78 million results in an estimated 150,340 (95% CI = 128,060, 173,060) people aged ≥18 years living with chronic HCV infection. Because this sample did not include incarcerated or homeless persons, groups known to have high HCV prevalence [19–22], this survey likely underestimates the true HCV burden. Two risk factors measured in this survey were significant, independent predictors of anti-HCV positivity: reported history of IDU and reported receipt of a blood transfusion. However, half of anti-HCV positive participants reported neither exposure, illustrating that screening based on reported risk factors alone will be insufficient to identify most chronically infected persons and eliminate HCV.

Communication about HCV transmission modes and disease course will be important components of efforts to increase screening. Half of all participants were unaware that they could have an HCV infection without experiencing any symptoms, and half were unaware that HCV is transmitted through exposure to infected blood. HCV-related knowledge was highest among participants reporting a history of IDU, possibly due to familiarity with the risks of injecting drugs. Although media coverage of the HCV elimination program within Georgia has likely increased the general public’s knowledge about HCV since this survey, these findings highlight the need to further intensify public education efforts to drive screening, particularly in groups less familiar with HCV transmission risks such as injecting drugs. However, identifying effective messaging and modes of communication could be challenging, given that only one-third of participants expressed trust in healthcare professionals as sources of health-related information, and even fewer reported trust in other sources including friends, family, radio, television, or the internet.

History of IDU was the strongest predictor of HCV infection in this survey and was reported by 38.2% of anti-HCV positive participants. IDU was most common among men, likely driving the three-fold difference in anti-HCV prevalence between men vs. women. In particular, men ages 40–49 years had the highest prevalence of both reported IDU (17.4%, data not shown) and anti-HCV (22.7%). (This cohort came of age during a drug trafficking and IDU epidemic in Georgia during the 1990s/early 2000s following the collapse of the former Soviet Union [23]). However, injecting behavior poses an important challenge for HCV elimination regardless of the age of persons injecting, and those actively injecting drugs will be a key target to curb transmission. Increasing access to harm reduction programs, including needle and syringe programs and medication for opioid use disorder, will be essential. In addition, a follow-up study among persons actively injecting drugs would further clarify HCV prevalence and risk behaviors in this sub-group to guide prevention efforts.

History of a blood transfusion also emerged as an independent risk factor for HCV infection and was reported by 20% of anti-HCV positive participants. Although Georgia began testing its donated blood supply for HCV in 1997, there was no detectable difference in anti-HCV prevalence between participants who received a transfusion before vs. after the blood testing program began. To halt HCV transmission and support elimination, it is imperative that Georgia evaluate and improve its blood safety program.

Nearly half of anti-HCV positive participants reported neither IDU nor blood transfusion. Possible explanations include underreporting of risk factors due to stigma, legal concerns, and poor recall, as well as HCV transmission through exposures not identified as potential risk factors in this survey. Suboptimal infection control during healthcare and dental procedures has been hypothesized as an HCV transmission risk in Georgia due to privatization and regulatory challenges in these sectors following the dissolution of the former Soviet Union. However, the cross-sectional nature of this survey and the near-universal utilization of dental and healthcare services make risk association difficult to detect from these exposures. Nonetheless, these data indicate that nearly half of HCV-infected persons in Georgia could be unaware of their risk history or unwilling to report it. Thus, screening strategies beyond a risk-based approach will be necessary for Georgia to identify enough infected persons to reach its elimination targets. In addition, further investigation is warranted to better understand potential HCV transmission risks in Georgia aside from IDU and blood transfusions, as well as differences in risk factors by sex.

Over 60% of participants with evidence of HCV infection learned about their status for the first time through participation in this survey. Among those already aware of their HCV infection, approximately one-third reported prior treatment; most would have been treated with interferon-based regimens, which were the only HCV treatment options available in Georgia prior to the launch of the national elimination program, and were cost-prohibitive for most Georgians. By offering DAA-based treatment to patients at no cost, Georgia’s HCV elimination program has addressed the primary treatment barriers cited by survey participants - expense, availability, and anticipated side effects. From the beginning of the elimination program in April 2015 through December 2016, 27,595 persons initiated treatment, and efforts are ongoing to continue to improve access for those who are aware of their HCV infection [18, 24]. With treatment infrastructure now in place, the greatest opportunity to boost progress toward HCV elimination lies in screening and diagnosing more infected individuals.

This survey has several limitations. Its cross-sectional design limits the ability to draw causal associations between possible exposures and HCV, a chronic infection that could have been acquired at any time before the survey. Further, the necessary reliance on self-reported risk factor data could result in information bias that is unmeasurable. The fact that IDU is illegal in Georgia and is the leading reason for incarceration [25] likely discourages self-reports of injecting behavior; similarly, high levels of MSM stigmatization likely explain the complete absence of self-reported MSM among participants in this survey. Finally, HCV prevalence among participants reporting a history of IDU at some point in their lifetime may not reflect HCV prevalence among persons actively injecting drugs, due to changes in infection dynamics in injecting populations over time.

## Conclusions

Georgia is working toward ambitious HCV elimination goals, aiming to screen and diagnose 90% of the estimated 150,000+ Georgians with chronic HCV infection, treat 95% of those identified, and reduce national prevalence of chronic HCV by 90% by 2020 [18]. This survey has provided nationally representative data to guide Georgia’s comprehensive HCV elimination strategy, as well as baseline HCV prevalence to evaluate progress toward HCV elimination in the coming years. In addition, conducting the survey provided an important opportunity to strengthen Georgia’s public health capacity and thereby enhance global health security

## Abbreviations

anti-HCV: antibodies to hepatitis C virus
AOR: adjusted odds ratio
CDC: United States Centers for Disease Control and Prevention
CI: confidence interval
DAA: direct-acting antiviral
Geostat: Georgia National Statistics Office
HCV RNA: hepatitis C virus ribonucleic acid
HCV: hepatitis C virus
IDU: injection drug use
MSM: men who have sex with men
NCDC: Georgia National Center for Disease Control and Public Health
